# A computational approach to distinguish somatic vs. germline origin of genomic alterations from deep sequencing of cancer specimens without a matched normal

**DOI:** 10.1371/journal.pcbi.1005965

**Published:** 2018-02-07

**Authors:** James X. Sun, Yuting He, Eric Sanford, Meagan Montesion, Garrett M. Frampton, Stéphane Vignot, Jean-Charles Soria, Jeffrey S. Ross, Vincent A. Miller, Phil J. Stephens, Doron Lipson, Roman Yelensky

**Affiliations:** 1 Foundation Medicine, Inc., Cambridge, MA, United States of America; 2 Institut National de la Santé et de la Recherche Médicale (INSERM) U981, Gustave Roussy, Villejuif Grand, Paris, France; 3 Oncology and Hematology Department, Hôpitaux de Chartres, Chartres, France; 4 Albany Medical College, Albany, NY, United States of America; Fox Chase Cancer Center, UNITED STATES

## Abstract

A key constraint in genomic testing in oncology is that matched normal specimens are not commonly obtained in clinical practice. Thus, while well-characterized genomic alterations do not require normal tissue for interpretation, a significant number of alterations will be unknown in whether they are germline or somatic, in the absence of a matched normal control. We introduce SGZ (somatic-germline-zygosity), a computational method for predicting somatic vs. germline origin and homozygous vs. heterozygous or sub-clonal state of variants identified from deep massively parallel sequencing (MPS) of cancer specimens. The method does not require a patient matched normal control, enabling broad application in clinical research. SGZ predicts the somatic vs. germline status of each alteration identified by modeling the alteration’s allele frequency (AF), taking into account the tumor content, tumor ploidy, and the local copy number. Accuracy of the prediction depends on the depth of sequencing and copy number model fit, which are achieved in our clinical assay by sequencing to high depth (>500x) using MPS, covering 394 cancer-related genes and over 3,500 genome-wide single nucleotide polymorphisms (SNPs). Calls are made using a statistic based on read depth and local variability of SNP AF. To validate the method, we first evaluated performance on samples from 30 lung and colon cancer patients, where we sequenced tumors and matched normal tissue. We examined predictions for 17 somatic hotspot mutations and 20 common germline SNPs in 20,182 clinical cancer specimens. To assess the impact of stromal admixture, we examined three cell lines, which were titrated with their matched normal to six levels (10–75%). Overall, predictions were made in 85% of cases, with 95–99% of variants predicted correctly, a significantly superior performance compared to a basic approach based on AF alone. We then applied the SGZ method to the COSMIC database of known somatic variants in cancer and found >50 that are in fact more likely to be germline.

## Author summary

We introduce SGZ, a computational method for predicting somatic vs. germline origin and homozygous vs. heterozygous or sub-clonal state of variants identified from deep massively parallel sequencing of clinical formalin-fixed, paraffin embedded (FFPE) cancer specimens. The method does not require fresh tissue or a patient matched normal control, enabling broad application in clinical research. It supports functional prioritization and interpretation of alterations discovered on routine testing and may inform clinical decision making and ultimately expand treatment choices for cancer patients.

This is a *PLOS Computational Biology* Methods paper.

## Introduction

Characterization of clinical cancer specimens using MPS for targeted treatment selection is becoming increasingly common [1–5]. These procedures generate large numbers of alterations per patient, only a minority of which are potential oncogenic drivers or therapeutically relevant, while the rest are either passenger mutations or germline polymorphisms that are typically functionally benign [6]. Although most therapeutic strategies will focus on variants that have already been well-characterized in the literature, an important opportunity to discover novel oncogenic targets will arise as hundreds of thousands of clinical cancer cases are sequenced. An essential component in this on-going analysis will be prioritizing uncharacterized variants for further follow-up, with somatic versus germline origin determination being a critical step.

The definitive approach to distinguishing somatic mutations from germline variants requires sequencing the tumor alongside a patient matched normal, and subsequently performing a comparison: variants detected in tumor tissue but not present in the normal control are advanced as mutation candidates [7–10]. However, while it is possible to establish protocols for paired collection in the academic cancer center setting, sequencing a patient matched normal specimen is not part of broad oncology practice, and known cancer drivers targetable by approved or investigational therapies can usually be discerned from tumor sequencing alone from well-established databases such as COSMIC [11]. It is therefore likely that as clinical cancer sequencing becomes routine and wide-spread, matched normal data will not be available for the majority of cases, foreclosing a significant opportunity for novel discovery and potential future therapeutic benefit unless this limitation is overcome. Although methods have been developed to determine germline status by matching to public germline databases like dbSNP or sequence a large number of normal individuals to be surrogates for matched normal [12], such methods cannot adequately account for rare germline variants that are private to a family or small population.

**Fig 1:**
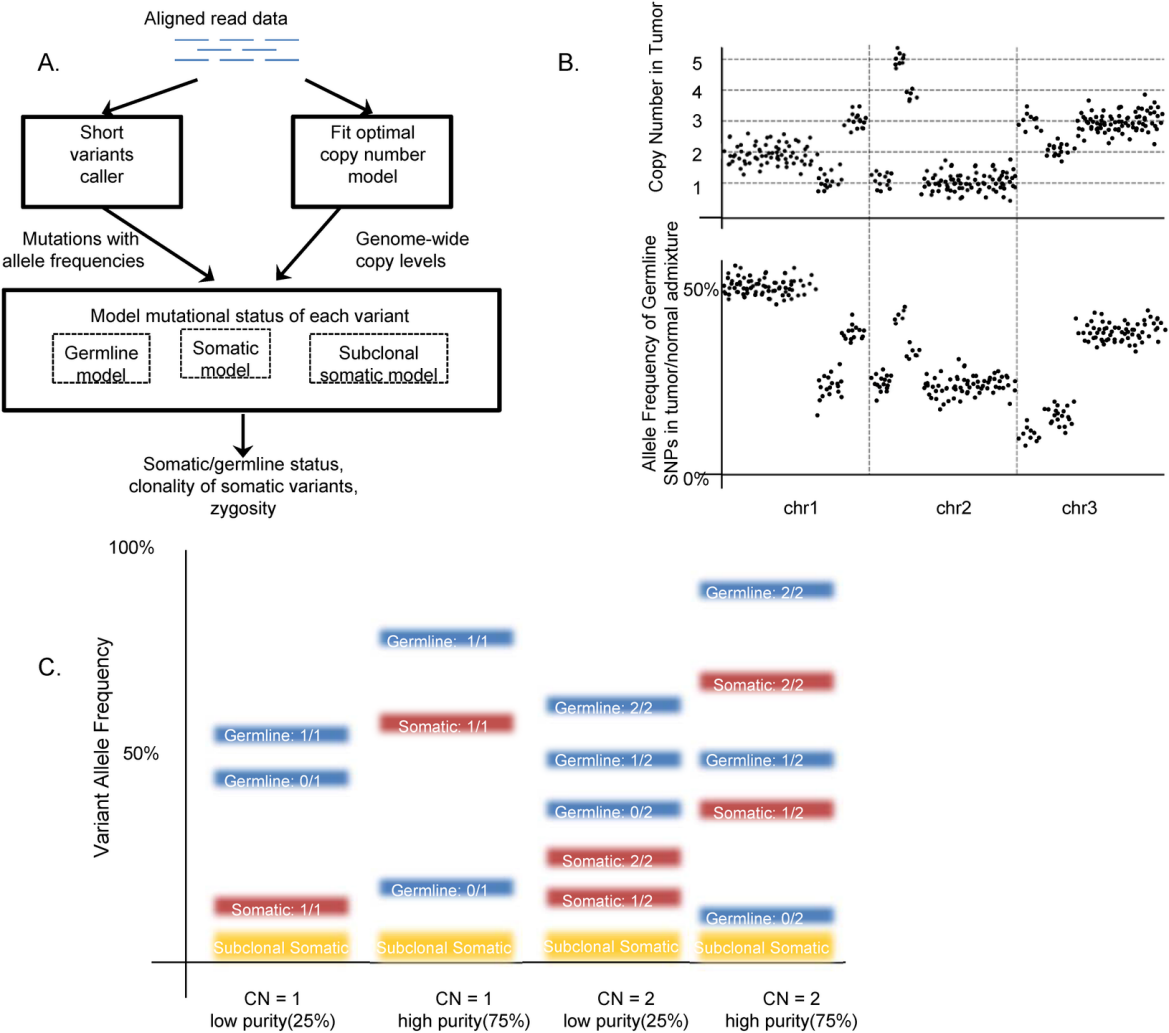
SGZ method overview. The SGZ pipeline is overviewed in panel A. Key components include fitting an optimal copy number model to the genome‐wide log‐ratio and minor allele frequency profiles (B), and modeling the expected allele frequencies of germline, somatic, and subclonal somatic mutations (C). In panel B, the dots in the top panel correspond to log ratios at each exon sequenced, segmented and fitted to discrete copy number levels, while the dots on the bottom panel are germline SNP minor allele frequencies. In panel C, examples of expected variant allele frequencies are shown for various scenarios of copy number and tumor purity. The expected allele frequencies are shown for germline (blue), somatic (red), and subclonal somatic (yellow).

We present SGZ, a novel computational method for predicting the somatic vs. germline origin of variants discovered in cancer specimens (Fig 1) without the need for a matched normal sample. In this method, the cancer specimen is sequenced to high depth (>500x) using MPS, in our implementation by a targeted clinical assay of 394 cancer-related genes and over 3,500 genome-wide SNPs [1]. SGZ leverages the precise measurement of the allele frequencies of variants of interest offered by deep sequencing and a statistical model of genome-wide copy number and tumor/normal admixture to characterize the mutational state of the variants. The method is generally applicable to any MPS sequencing platform where the sequencing depth is sufficient, an accurate model of copy number can be created, and the tumor specimen is sufficiently admixed with the surrounding normal tissue.

## Methods

**Fig 2:**
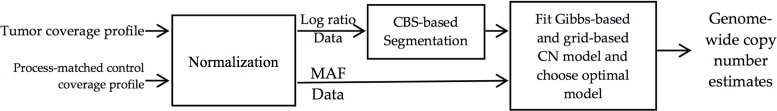
Copy number detection overview. Aligned DNA sequences of the tumor specimen are normalized against a process‐matched normal, producing log‐ratio and minor allele frequency (MAF) data. Next, whole‐genome segmentation is performed using a circular binary segmentation (CBS) algorithm on the log‐ratio data. Then, a Gibbs sampler fitted copy number model and a grid‐based model are fit to the segmented log‐ratio and MAF data, producing genome‐wide copy number estimates. Finally, the degree of fit of candidate models returned by Gibbs sampling and grid sampling are compared and the optimal model is selected by an automated heuristic.

The SGZ method works as follows (Fig 1, S1 Fig): For each sample, we first execute a standard MPS variant analysis pipeline, which aligns unique sequence reads and obtains candidate mutations with associated mutant allele frequencies [1]. The pipeline also creates a genome-wide copy number profile based on coverage and allele frequencies at over 3,500 SNPs, which is segmented and modeled to estimate the overall tumor purity (*p*) and ploidy (Ψ), as well as the per segment copy number (*C*) and minor allele count (*M*). An overview of our copy number detection approach is shown in Fig 2. To obtain a log-ratio profile of signal intensity, aligned tumor sequence reads are normalized by dividing read depth by that of a process-matched normal control, followed by a GC-content bias correction using Lowess regression. The minor allele frequency (MAF) profile is obtained from the heterozygous genome-wide SNPs. These constitute the observed data for the statistical model.

We fit the log-ratio and MAF data by a statistical model which predicts genome-wide copy number profile. This is done in two steps: First, we use the circular binary segmentation (CBS) algorithm to divide the genome into segments of equal copy number [13]. CBS recursively divides the log-ratio data into individual segments until each segment is homogenous such that no further divisions lead to statistically significant differences in signal level. Depending on the aneuploidy and data quality of one sample, the number of segments can range from 22 to a few hundred. Second, we use the segment-based log-ratio and MAFs to fit the statistical copy number model. Briefly, if *S*_*i*_ is a genomic segment, let *l*_*i*_ be its length and *C*_*i*_ be its copy number. The tumor ploidy Ψ of the sample is $\Psi =\frac{\sum_{i}l_{i}C_{i}}{\sum_{i}l_{i}}$. If *r*_*i*_ is the random variable representing the median-normalized log-ratio coverage of all exons within *S*_*i*_, and *p* is the tumor purity, we model *r*_*i*_ as a Normal distribution as:

$$r_{i}∼N(\log_{2}\frac{pC_{i}+2(1-p)}{p\Psi +2(1-p)},\sigma_{ri})$$ (1)

where *σ*_*ri*_ is the SD of the log-ratio data in segment *S*_*i*_, reflecting the noise observed. Similarly, if *f*_*i*_ random variable represents the MAF of SNPs within segment *S*_*i*_, *M*_*i*_ the copy number of minor alleles in *S*_*i*_, distributed as integer 0 ≤ *M*_*i*_ ≤ *C*_*i/2*_, and *σ*_*fi*_ the SD of the SNP data at segment *S*_*i*_, we model *f*_*i*_ as:

$$f_{i}∼N(\frac{pM_{i}+1-p}{pC_{i}+2(1-p)},\sigma_{fi})$$ (2)

Given this model of the log-ratio and MAF, a two-step approach is used to find the optimal fit of model parameters *C*_*i*_ and *M*_*i*_ at each segment, as well as the genome-wide model parameters tumor purity (*p*) and ploidy (Ψ). First, an initial fit is assessed using the JAGS software package [14], a Gibbs sampling based Markov Chain Monte Carlo algorithm. Assuming a sample has 200 segments after segmentation, the total number of parameters is more than 400. Based on our pipeline design, there are around 10,000 observed SNPs and 50,000 observed median-normalized log-ratios. After checking the convergence of all parameters, the following key MCMC parameters are employed: sampling size at 500, burn-in size at 500, thinning interval at 1 and 9 chains. Second, a grid-based method is used to find alternative solutions that can also fit the model [15]. The grid-based method evaluates the mean-squared-error between the measured and the expected copy numbers, over a grid of different tumor purity and ploidy. All local minima in the grid are considered as model candidates.

The goodness of fit of all copy number models returned by Gibbs sampling and grid sampling are assessed by the mean squared error (MSE) of log-ratios of all segments and the MSE of MAF of all segments. Gibbs model is the default optimal model and is compared to grid-based copy number models at the first three local minima. A grid-based copy number model is selected as the final optimal model if it is proven to meet all of the following five requirements: 1) the MSE of log-ratios and the MSE of MAFs are reduced; 2) the ploidy is higher than 1.2; 3) the model does not have excessive copy number loss events (CN = 0); 4) it is not a more complex model, which is defined by a higher ploidy delta of at least 1.1 and a lower purity delta of at least 0.1; 5) it is not a high purity sample (predicted purity > 0.99) unless an independent high purity estimation prediction algorithm agrees.

Given the output of the copy number model, each variant’s measured AF is compared to expectation at its local segment *i*: ${AF}_{germline}=\frac{pV_{i}+1-p}{pC_{i}+2(1-p)}$ vs. $AF_{somatic}=\frac{pV_{i}}{pC_{i}+2(1-p)}$, where *V*_*i*_ is the variant allele count in the tumor, which can be either *M*_*i*_ or *C*_*i*_*-M*_*i*_. To determine whether a variant is predicted somatic, germline, or ambiguous, we used the following statistical model: Define *y*: *= (n*,*f)*, where *y* is the variant data comprising read depth *n* and allele frequency *f*; G: = germline hypothesis; and S: = somatic hypothesis. Given the germline hypothesis G, the probability of *y* is obtained using the 2-tailed binomial test *P*(*y*|*G*; *AF*_*germline*_) = *Bin* (*nf*, *n*, *AF*_*germline*_). Given the somatic hypothesis S, the probability of *y* is obtained using the 2-tailed binomial test *P*(*y*|*S*; *AF*_*somatic*_) = *Bin* (*nf*, *n*, *AF*_*somatic*_). A variant is predicted somatic if *P*(*y*|*S*; *AF*_*somatic*_) > *α* and *P*(*y*|*G*; *AF*_*germline*_) ≤ *α*. A variant is predicted germline if *P*(*y*|*S*; *AF*_*somatic*_) ≤ *α* and *P*(*y*|*G*; *AF*_*germline*_) > *α*. A variant is predicted subclonal somatic if *P*(*y*|*S*; *AF*_*somatic*_) ≤ *α*, *P*(*y*|*G*; *AF*_*germline*_) ≤ *α*, and *f* < *AF*_*somatic*_ / 1.5. Subclonal somatic predictions are made only in samples with a tumor purity of greater than 20%. A variant is declared ambiguous and not called if none of the conditions above holds. The variable *α* is set to be 0.01. All possible prediction outcomes are enumerated in S2A Fig, with an example sample shown in S2B Fig.

Similar to prior studies [15–18], the SGZ method classifies the tumor zygosity of the mutation (homozygous vs. heterozygous) or predicts that the mutation resides in a minor subclone. A variant in the tumor is classified as homozygous if all copies in the tumor carries the mutant allele (*V = C* and *V≠0*), heterozygous if both the reference and the mutant are present (*V≠C* and *V≠0*), and not in tumor if the tumor only carries the reference (*V = 0*, applicable only to germline variants). A somatic mutation is further classified as subclonal if the allele frequency is significantly less than the lowest expected allele frequency.

## Results

### Method validation datasets

We validated SGZ in three different ways, including (1) specimens with matched normal where the true origin of all alterations was known, (2) cell-line admixtures that modeled the impact of varying tumor purity on the inference, and (3) a large set of clinical FFPE specimens with known somatic drivers where real-world somatic variant recovery was assessed.

The first dataset consisted of 87 specimens from 30 non-small cell lung and colon cancer patients, wherein each patient we studied three samples: the primary tumor, a metastatic site, and adjacent tissue matched normal (S3 Table). All DNA were extracted from fresh-frozen clinical specimen. The primary and metastatic tumors uniformly contained a mixture of malignant and benign epithelial, stromal and inflammatory cells. The gold standard origin of a mutation is established by following the rules: whenever a variant appeared in the matched normal with significant allele frequency, it was considered germline, and tumor-only variants were called somatic. In several samples, low levels of tumor infiltrated into the matched normal sample, hence the sample was found to carry low levels of mutation with allele frequency <10%. These were regarded as somatic mutations. A total of 330 unique variants were detected and evaluated, including 70% (N = 231) germline and 30% (N = 99) somatic according to gold standard. SGZ was applied to the primary and metastatic tumor samples to make somatic/germline predictions. DNA from the 30 non-small cell lung and colon cancer patients was obtained from Institute Gustave Roussy [19, 20].

To assess the robustness of the method to different levels of tumor purity, we examined three cancer cell lines (HCC-1937, HCC-1954, & NCI-H1395), which were titrated with their matched lymphoblastoid normal to six levels of tumor purity (10%, 20%, 30%, 40%, 50%, 75%). A total of 42 unique variants were detected by our pipeline and used for validation (S4 Table).

The third dataset is data from 20,182 clinical FFPE tissue samples sent to Foundation Medicine for FoundationOne testing. The samples were of a variety of tumor types, originating from a wide diversity of cancer centers and community oncology practices. To evaluate SGZ predictions of germline/somatic origin, we examined predictions at 17 known somatic hotspot mutations (e.g. *BRAF* V600, *KRAS* G12) and 20 common germline SNPs. To assess SGZ predictions of tumor zygosity, we selected the most frequently mutated somatic variants at oncogenes (*BRAF*, *EGFR*, *IDH1*, *KRAS*, *NRAS*, *PIK3CA*) and tumor suppressor genes (*TP53*, *RB1*, *PTEN*) for analysis. To assess the ability of SGZ to detect subclonal mutations, we examined *EGFR* T790M, a common subclonal tyrosine kinase inhibitor resistance mutation, in all the non-small cell lung samples in this dataset (N = 69). The FoundationOne assay platform, its clinical application, and an early description of the cohort genomics is described in Frampton et al. 2013.

### Method validation results

To demonstrate the importance of taking into account the genome-wide copy number profile for somatic/germline prediction, we applied SGZ to the three validation datasets and compared SGZ to a method that does not take tumor aneuploidy into account (referred to as “basic method”), in which a variant is classified as germline if its mutation frequency is near 50% or 100%, or otherwise is classified as somatic [21] (S1 Method).

SGZ yielded somatic vs. germline calls for 85% of variants in the lung and colon samples, 83% of variants in the three cell lines admixtures, and 84% in the 17 somatic hotspot mutations and 20 common germline variants in the 20,182 Foundation Medicine clinical samples. Among these calls, 95%, 97% and 96% of the somatic mutations were predicted correctly, respectively; 99%, 97%, and 97% of the germline mutations were predicted correctly, respectively. On the contrary, the basic method was able to make predictions for 100% of the variants in the three datasets, but only predicted somatic variants correctly 67%, 92% and 95% of time, and germline variants correctly 87%, 41% and 51% of the time, which are significantly lower than the accuracy of SGZ. Importantly, in none of the three datasets did the basic method achieve satisfactory performance in both germline mutations and somatic mutations simultaneously (Table 1, S1 Table). In the cell line dataset, out of a total number of 184 short variants that are correctly classified by SGZ, 63 short variants are incorrectly classified by the basic method due to local copy number deviation from 2 and/or zygosity deviation from the heterozygous state, strongly suggesting the necessity to take copy number variation into account in order to make accurate predictions (S5 Table, S3 Fig).

**Table 1:** Validation of somatic and germline predictions.

Validation study	Call rate	Somatic variants predicted correctly	Germline variants predicted correctly
All variants in 30 lung & colon samples with matched-normal as gold standard (**basic method**)	100% (568/568)	67% (255/380)	87% (164/188)
All variants in 30 lung & colon samples with matched-normal as gold standard (**SGZ**)	85% (480/568)	95% (312/327)	99% (151/153)
All variants in 3 cell lines with varying proportions of tumor-normal admixture (**basic method**)	100% (215/216)	92% (83/90)	41% (51/125)
All variants in 3 cell lines with varying proportions of tumor-normal admixture (**SGZ**)	83% (184/222)	97% (60/62)	97% (118/122)
17 somatic hotspot mutations and 20 common germline variants in 20,182 clinical samples (**basic method**)	100% (12506/12506)	95% (7213/7560)	51% (2537/4946)
17 somatic hotspot mutations and 20 common germline variants in 20,182 clinical samples (**SGZ**)	84% (9829/11646)	96% (5325/5540)	97% (4172/4289)

**Fig 3:**
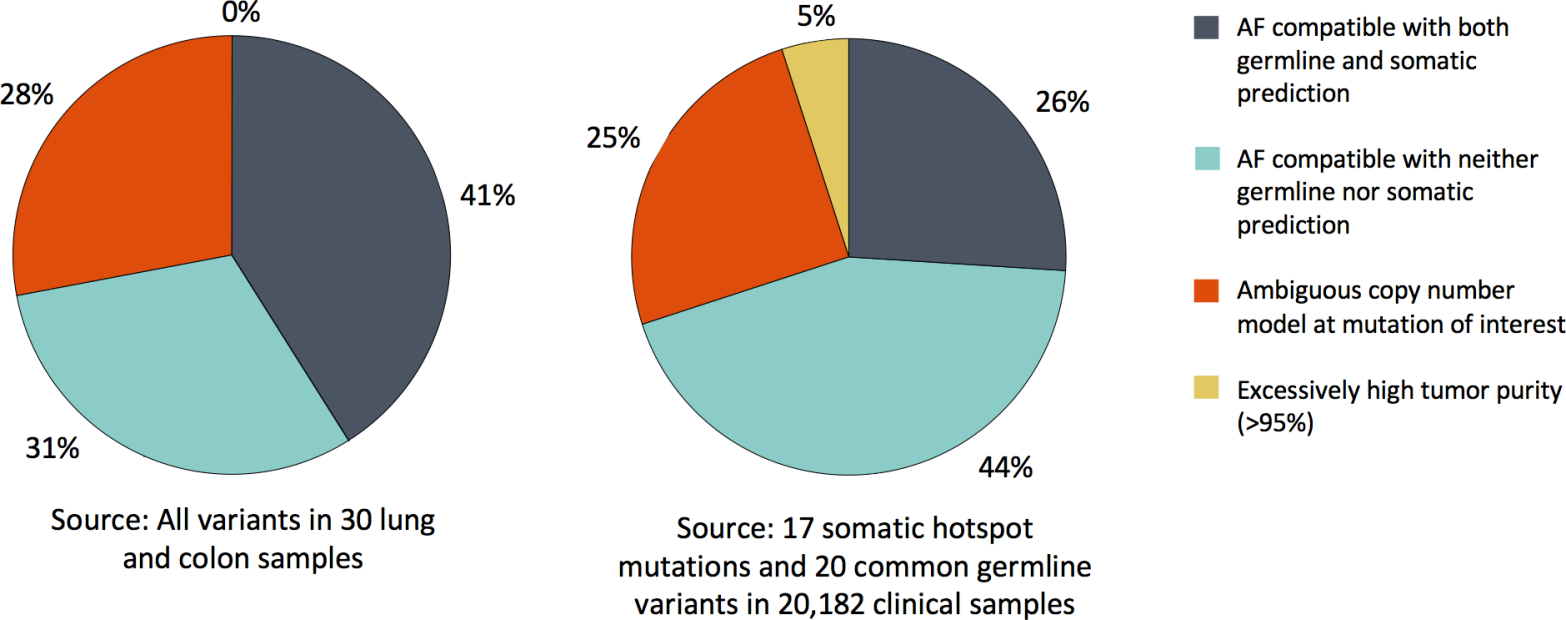
Breakdown of no-calls made by SGZ. Reasons behind no-calls made by SGZ are shown for (left) all variants in 30 lung and colon samples and (right) 17 somatic hotspot mutations and 20 common germline variants within 20,182 clinical samples.

SGZ had a no-call rate in around 15% of mutations in the lung and colon samples and the Foundation Medicine clinical dataset due to multiple factors (Fig 3), including excessively high tumor purity (>95%), gross deviations of the copy number model at the variant site, observed mutation AF compatible with both somatic and germline AF expectations, and observed AF outside of both somatic and germline expectations.

To characterize the performance of SGZ as a function of tumor purity, we captured the call rate and prediction accuracy of SGZ in each tumor purity level in the cell-line dataset (Table 2). Overall, the call rate is between 75% to 94%, and the prediction accuracy ranges from 88% to 100%. As expected, the call rate at 10% tumor purity is the highest among all dilution levels, due to the large difference between expected germline and somatic AF (S1 Fig). Though not available in this dataset, it is expected that call rate would rapidly drop to 0% as the tumor purity exceeds 90% due to much smaller differences between somatic and germline variant AF expectations. For germline and somatic prediction accuracy, a high level of accuracy is maintained from 10% through 75% tumor purity. It is also expected that the prediction accuracy would drop as tumor content exceeds 90%.

**Table 2:** SGZ performance as a function of tumor purity in the cell line dataset.

Tumor Purity	10%	20%	30%	40%	50%	70%
Call Rate	0.94	0.89	0.83	0.78	0.75	0.80
Germline Accuracy	1.00	1.00	1.00	1.00	0.94	0.88
Somatic Accuracy	1.00	1.00	0.92	1.00	0.90	1.00

To assess SGZ predictions of tumor zygosity, we examined data from the most frequently mutated somatic variants at oncogenes (*BRAF*, *EGFR*, *IDH1*, *KRAS*, *NRAS*, *PIK3CA*) and tumor suppressor genes (*TP53*, *RB1*, *PTEN*) in the Foundation Medicine clinical sample set. Alterations in oncogenes are expected to be mostly heterozygous, as a single mutation is required for activation, whereas the tumor suppressor genes are expected to have the first functional copy inactivated via mutation, and the second inactivated through loss-of-heterozygosity (LOH) [22]. Our predictions of tumor zygosity are concordant with the roles these genes play: *TP53* and *RB1* were to determined have >90% of mutations under LOH, while *BRAF* V600 and *KRAS* G12 mutations showed no significant enrichment for LOH (Table 3).

**Table 3:** Tumor zygosity predictions of somatic mutations in 20,182 clinical samples.

Gene	Amino acid affected	Gene type	Samples with mutation	Mutations with LOH	LOH enrichment ratio^1^
*BRAF*	V600^†^	Oncogene	279	6.8%	0.61
*EGFR*	L858R	Oncogene	116	4.3%	0.63
*IDH1*	R132H	Oncogene	131	0.8%	0.06
*KRAS*	G12^†^	Oncogene	1444	16.6%	1.21
*NRAS*	Q61^†^	Oncogene	198	13.1%	0.68
*PIK3CA*	H1047^†^	Oncogene	347	11.5%	0.86
*PTEN*	All substitutions^‡^	Suppressor	308	81.8%	3.54
*RB1*	All substitutions^‡^	Suppressor	307	90.6%	2.75
*TP53*	All substitutions^‡^	Suppressor	4666	91.8%	3.74

^1^The enrichment ratio with respect to background LOH percentage, which is measured in non-mutated samples at the genomic locations in each gene.

^†^Includes all missense mutations of the codon.

^‡^All missense and nonsense substitutions of confirmed somatic status in COSMIC or consensus splice site variants. Samples with compound heterozygous mutations in a gene are excluded as they are not expected to be under LOH.

To assess our ability to detect subclonal mutations, we examined *EGFR* T790M in the non-small cell lung carcinoma subset of our dataset, where the mutation would be expected to occur in tyrosine kinase inhibitor resistant subclones. Indeed, we discovered a significant enrichment of subclonal somatic vs. somatic heterozygous/homozygous calls for T790M –a ratio of 1.5 (41/28)–compared to a ratio of only 0.24 (1043/4282) for the 17 somatic hotspot mutation sites. The SGZ method was also applied to predict the clonality and zygosity of 12 *ESR1* mutations in estrogen receptor positive breast cancer biopsies and determined these *ESR1* mutations to be somatically acquired, clonal biomarkers of endocrine resistance [23].

### Cancer database application

Despite best efforts of cancer investigators leveraging matched normal controls, germline variants may erroneously get nominated and recorded as somatic mutations in the literature and public catalogues of somatic variation, due to the challenges inherent in large scale sequencing studies and MPS data analysis. These variants may divert scarce resources needed for functional follow-up or potentially mislead therapeutic choice if pursued clinically. It would thus be beneficial if these false somatic variants could be collectively flagged and potential interpretation and application corrected.

To discover mutations that may be misclassified as somatic in a public database, we applied the SGZ method to the 20,182 clinical specimens to identify variants predicted to be germline but annotated in COSMIC (v62) as somatic. To confidently call a variant as germline, we required germline predictions in multiple specimens and obtained p-values using a binomial model of SGZ error rate by tabulating the number of somatic, germline, and ambiguous predictions for each variant and obtaining *P*(*S*|*n*_*G*_, *n*_*S*_), the probability of a variant being somatic, given the *n*_*G*_ germline calls and *n*_*S*_ somatic calls: Using Bayes rule and a flat prior, i.e *P*(*G*) = *P*(*S*) = 0.5, $P\left(S|n_{G},n_{S}\right)=\frac{P\left(n_{G},n_{S}|S\right)}{P\left(n_{G},n_{S}|G\right)+P(n_{G},n_{S}|S)}$. Multiple observations were modeled as binomial distributions: $P\left(n_{G},n_{S}|G\right)=\left(\begin{array}{c} n_{G}+n_{S}\\ n_{G} \end{array}\right)e_{G}^{n_{S}}{\left(1-e_{G}\right)}^{n_{G}}$ and $P\left(n_{G},n_{S}|S\right)=\left(\begin{array}{c} n_{G}+n_{S}\\ n_{S} \end{array}\right)e_{S}^{n_{G}}{\left(1-e_{S}\right)}^{n_{S}}$ with *e*_*G*_ as the single sample germline error rate, i.e. the probability of SGZ making an error given a germline prediction is made and *e*_*S*_ as the single sample somatic error rate. We used conservative parameters *e*_*G*_ = 0.05 and *e*_*S*_ = 0.10, which are higher than the error rates from Fig 2A. *P*(*G*|*n*_*G*_, *n*_*S*_) can be readily obtained as 1 – *P*(*S*|*n*_*G*_, *n*_*S*_).

Table 4 shows the top 10 variants present in COSMIC, but strongly predicted by our method as germline. Each variant was predicted germline in at least 45 samples. Although 9 of 10 variants were annotated as confirmed somatic, the number of entries in the database were all low (≤4), reinforcing that the somatic annotation is likely inaccurate. Further evidence of germline origin was that most variants had an entry in dbSNP, though few were classified as common SNPs. The full list of seventy COSMIC variants predicted to be germline is given in S2 Table.

**Table 4:** Likely somatic status mis-annotation in COSMIC, predicted by SGZ to be germline in multiple samples in Foundation Medicine sample set^†^.

Gene	Protein change	Status in COSMIC v62	Entries in COSMIC	dbSNP ID	Common SNP in 1000 Genomes	P-value^‡^
*EP300*	P925T	Confirmed somatic	1	rs148884710	No	8.0E-235
*VHL*	P25L	Confirmed somatic	1	rs35460768	No	3.0E-191
*CSF1R*	V32G	Confirmed somatic	1	rs56048668	No	3.4E-181
*APC*	I1307K	Confirmed somatic	1	rs1801155	No	1.5E-159
*RET*	Y791F	Confirmed somatic	1	rs77724903	No	6.4E-124
*MSH6*	V509A	Confirmed somatic	1	rs63751005	No	3.0E-84
*MLL*	L3614P	Confirmed somatic	1	rs146191865	Yes	7.6E-71
*IL7R*	T244I	Confirmed somatic	2	rs6897932	Yes	2.3E-60
*CREBBP*	S893L	Confirmed somatic	4	rs142047649	No	5.7E-47
*ATM*	S978P	Unknown	1	rs139552233	No	2.0E-45

^†^The listed mutations have “confirmed somatic” status in COSMIC, but are likely mis-annotation, as the number of references supporting the status is low, while SGZ predicted these variants to be germline in multiple samples. Furthermore, although the mutations are not necessarily common SNPs, each mutation has a dbSNP entry, which further supports germline status.

^‡^Probability of being somatic, given multiple SGZ predictions for each variant.

## Discussion

The SGZ method leverages deep MPS to predict variant somatic and germline origin without a matched normal control. While the definitive approach for discovery of novel somatic mutations includes sequencing a patient matched normal, SGZ supports functional prioritization and interpretation of alterations discovered on routine testing performed with tumor alone and can enable assay development and clinical research.

There are several limitations of the SGZ method. Samples must have adequate admixture of the surrounding normal tissue. The exact mixture requirement depends on sequencing depth, which is considered in our statistical model on a per mutation basis, but given our coverage depth of >500X, somatic versus germline calling generally requires at least 10% normal tissue, i.e. tumor content under 90%. This held for 97% of solid tumor clinical cancer specimens that we sequenced. Zygosity calling required estimated tumor purity to be at least 20%, which held for 76% of our sample set.

Accuracy of the copy number model is likewise important. Minor misfit of the model can lead to an elevated rate of no calls, and major misfit of the model can lead to misclassification of somatic versus germline status, especially when tumor content is high, where the expected difference between germline and somatic allele frequency is reduced (S1 Fig). However, in copy number modeling, a key subset of copy number models is mathematically equivalent in terms of SGZ predictions, which improves robustness (S1 Note). Additionally, in low tumor content samples, the differences in expected allele frequencies between germline and somatic mutations are large, hence more robust to deviations in copy number model.

As shown in S1 Fig, there are also limited scenarios where the differences in expected allele frequencies between germline and somatic mutations are small, hence a prediction cannot be made. For example, a mutation with measured allele frequency of 33% in a genomic region with copy number 3 and LOH is equally likely to be either “germline and not in tumor” or “somatic and homozygous”. Finally, there is a scenario in which a subclonal somatic mutation produces an allele frequency equivalent to the expected germline frequency, misclassifying the mutation as germline. In practice, this is rare.

Despite these limitations, SGZ achieved impressive accuracy in validation studies, reaching call rates of 85% and accuracy of 95–99% when applied to individual samples. Importantly, for recurrent mutations (typical focus of cancer studies and clinical research), a key way to improve performance is to apply SGZ to a large cohort of samples, where recurrent mutations can be tabulated in the number of times a germline or somatic prediction is made. This information can be used to annotate variants for which somatic/germline status is unknown or in doubt. When applied over a large cohort of samples, SGZ can aid in the discovery of novel recurrent somatic mutations, along with their clonality and zygosity status [23, 24]. Conversely, SGZ can also identify germline variants not yet catalogued in public databases such as dbSNP and flag them from further consideration as cancer drivers. In this report, we describe the computational approach, which may be implemented on any cancer deep sequencing platform with copy number modeling support and provide both the methodology and a detailed worksheet to ease implementation (S1 Fig). We also apply the method to generate a proposed re-annotation of a large number of variants currently believed to be somatic, in the hope of improving the reliability of publicly availably cancer information. Ultimately, the application of SGZ may inform clinical decision making and expand treatment choices for cancer patients.

## Supporting information

S1 Method Basic somatic/germline prediction comparator method. (PDF)

S2 Method Finding COSMIC variants in dbSNP. (PDF)

S1 Fig Table of expected mutational allele frequencies. (PDF)

S2 Fig All possible SGZ prediction outcomes and an example of a cancer specimen across the genome. (PDF)

S3 Fig Exemplar high aneuploidy NCI-H1395 cell line with dilution with matched normal to 50% tumor purity to the advantage of SGZ using copy number model to make correct germline/somatic predictions, as compared to the basic method. (PDF)

S1 Table Somatic hotspot mutations and germline polymorphisms used for SGZ validation. (PDF)

S2 Table List of variants in COSMIC predicted to be germline. (PDF)

S3 Table Summary of 84 samples from 30 non-small cell lung and colon cancer patients. (PDF)

S4 Table Mutations from cell line dataset that were detected by our pipeline and used for SGZ validation. (PDF)

S5 Table Mutations incorrectly classified by the basic method and correctly classified by SGZ in regions of copy number change in the cell line dataset. (PDF)

S1 Note Equivalence of a subset of SGZ solutions to copy number model fitting. (PDF)
