# Supplementary material for: A computational approach to distinguish somatic vs. germline origin of genomic alterations from deep sequencing of cancer specimens without a matched normal
Source: PLoS Comput Biol. 2018 Feb 7;14(2):e1005965. doi: 10.1371/journal.pcbi.1005965 (PMC5832436; doi:10.1371/journal.pcbi.1005965)

S1 Method. Basic somatic/germline prediction comparator method. As suggested by Jones et al. 2015, the tumor-cellularity-based algorithm uses the fraction of mutational allele and the sample purity alone to distinguish germline from somatic mutations. Briefly, for a mutation with allele frequency f and depth n from a sample with purity p: 1) if $f > 0.95$ and $p < 0.90$, the mutation is reported as germline. If $p > 0.9$, it's classified as ambiguous due to high tumor content; 2) if $f < 0.95$, a binomial test is performed to assess the probability of mutational allele to be nf or further from $0.5f$, which is the p-value. If p-value < 0.05, the mutation will be reported as somatic and germline otherwise. The following flowchart visually shows the implementation of the basic tumor-cellularity-based algorithm to which we compare somatic/germline classification performance.

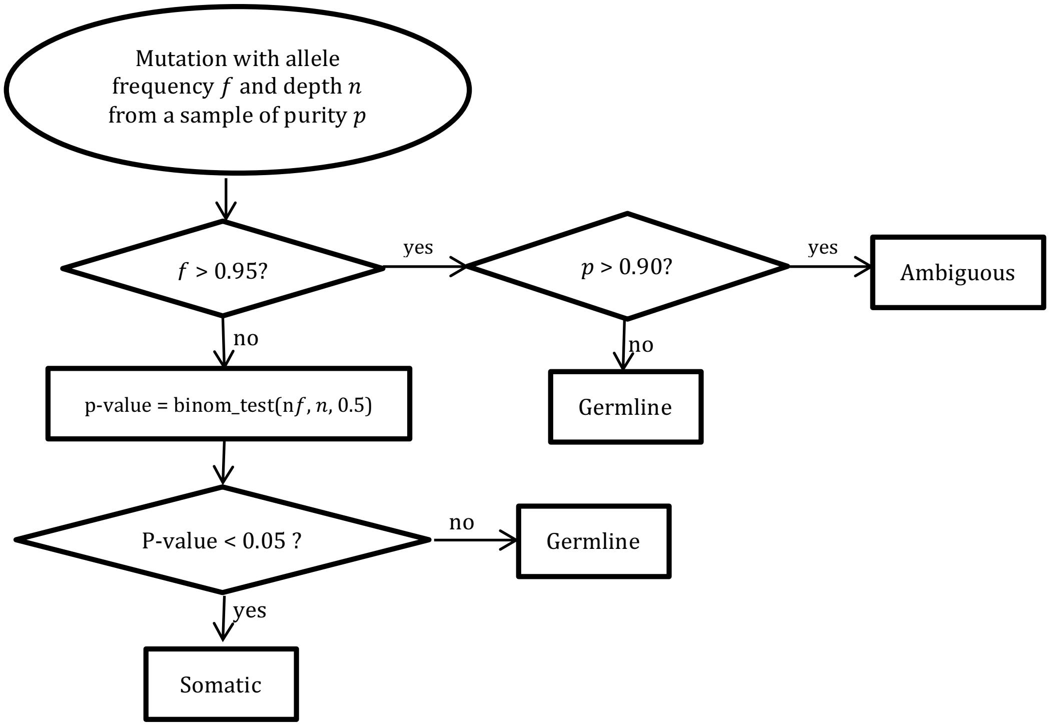

Supplement: S1 Method — (PDF) [file pcbi.1005965.s001.pdf]
