# Supplementary material for: A computational approach to distinguish somatic vs. germline origin of genomic alterations from deep sequencing of cancer specimens without a matched normal
Source: PLoS Comput Biol. 2018 Feb 7;14(2):e1005965. doi: 10.1371/journal.pcbi.1005965 (PMC5832436; doi:10.1371/journal.pcbi.1005965)
Supplement: S2 Method — (PDF) [file pcbi.1005965.s002.pdf]

S2 Method. Finding COSMIC variants in dbSNP. The rsIDs of COSMIC variants predicted to be germline were collected using build 141 of dbSNP, using the variants listed in the All.vcf available from dbSNP's FTP server (ftp://ftp.ncbi.nih.gov/snp/organisms/human_9606_b141_GRCh37p13/VCF/). Common SNPs were labeled in the All.vcf file, and were defined as SNPs with a minor allele frequency of at least 1% observed in two or more founders in any population studied in the 1000 Genomes Project.
