# Supplementary material for: A computational approach to distinguish somatic vs. germline origin of genomic alterations from deep sequencing of cancer specimens without a matched normal
Source: PLoS Comput Biol. 2018 Feb 7;14(2):e1005965. doi: 10.1371/journal.pcbi.1005965 (PMC5832436; doi:10.1371/journal.pcbi.1005965)
Supplement: S1 Fig — (PDF) [file pcbi.1005965.s003.pdf]

S1 Fig. Table of expected mutational allele frequencies. Given copy number (C), variant allele count (V), sample purity (p), and variant status (somatic or germline), we can generate a table of expected allele frequencies. V is the number of copies in a tumor cell that carries the mutation, C is the tumor number of copies for all variants at the same physical position, and p is the fraction of cells in the sequenced sample that is tumor. In the germline setting, the expected allele frequencies are computed as $AF_{germline} = \frac{pV+1-p}{pC+2(1-p)}$. In the somatic setting, it is $AF_{somatic} = \frac{pV}{pC+2(1-p)}$. These expected allele frequencies are enumerated below, for a sample of p, V, and C.

copy number	LOH status	status of variant	p: sample purity 0	5	10	15	20	25	30	35	40	45	50	55	60	65	70	75	80	85	90	95	100
C=1	LOH	V=0 germline	50	49	47	46	44	43	41	39	38	35	33	31	29	26	23	20	17	13	9	5	0
		V=1 somatic	0	3	5	8	11	14	18	21	25	29	33	38	43	48	54	60	67	74	82	90	100
		V=1 germline	50	51	53	54	56	57	59	61	63	65	67	69	71	74	77	80	83	87	91	95	100
C=2	LOH	V=0 germline	50	48	45	43	40	38	35	33	30	28	25	23	20	18	15	13	10	8	5	3	0
		V=2 somatic	0	5	10	15	20	25	30	35	40	45	50	55	60	65	70	75	80	85	90	95	100
		V=2 germline	50	53	55	58	60	63	65	68	70	73	75	78	80	83	85	88	90	93	95	98	100
	het	V=1 somatic	0	3	5	8	10	13	15	18	20	23	25	28	30	33	35	38	40	43	45	48	50
		V=1 germline	50	50	50	50	50	50	50	50	50	50	50	50	50	50	50	50	50	50	50	50	50
C=3	LOH	V=0 germline	50	46	43	40	36	33	30	28	25	22	20	18	15	13	11	9	7	5	3	2	0
		V=3 somatic	0	7	14	21	27	33	39	45	50	55	60	65	69	74	78	82	86	89	93	97	100
		V=3 germline	50	54	57	60	64	67	70	72	75	78	80	82	85	87	89	91	93	95	97	98	100
	het	V=1 somatic	0	2	5	7	9	11	13	15	17	18	20	22	23	25	26	27	29	30	31	32	33
		V=1 germline	50	49	48	47	45	44	43	43	42	41	40	39	38	38	37	36	36	35	34	34	33
		V=2 somatic	0	5	10	14	18	22	26	30	33	37	40	43	46	49	52	55	57	60	62	64	67
		V=2 germline	50	51	52	53	55	56	57	57	58	59	60	61	62	62	63	64	64	65	66	66	67
C=4	LOH	V=0 germline	50	45	41	37	33	30	27	24	21	19	17	15	13	11	9	7	6	4	3	1	0
		V=4 somatic	0	10	18	26	33	40	46	52	57	62	67	71	75	79	82	86	89	92	95	97	100
		V=4 germline	50	55	59	63	67	70	73	76	79	81	83	85	88	89	91	93	94	96	97	99	100
	het	V=1 somatic	0	2	5	7	8	10	12	13	14	16	17	18	19	20	21	21	22	23	24	24	25
		V=1 germline	50	48	45	43	42	40	38	37	36	34	33	32	31	30	29	29	28	27	26	26	25
		V=3 somatic	0	7	14	20	25	30	35	39	43	47	50	53	56	59	62	64	67	69	71	73	75
		V=3 germline	50	52	55	57	58	60	62	63	64	66	67	68	69	70	71	71	72	73	74	74	75
	het	V=2 somatic	0	5	9	13	17	20	23	26	29	31	33	35	38	39	41	43	44	46	47	49	50
		V=2 germline	50	50	50	50	50	50	50	50	50	50	50	50	50	50	50	50	50	50	50	50	50
C=5	LOH	V=0 germline	50	44	39	35	31	27	24	21	19	16	14	12	11	9	7	6	5	3	2	1	0
		V=5 somatic	0	12	22	31	38	45	52	57	63	67	71	75	79	82	85	88	91	93	96	98	100

	V=5	germline	50	56	61	65	69	73	76	79	81	84	86	88	89	91	93	94	95	97	98	99	100
het	V=1	somatic	0	2	4	6	8	9	10	11	13	13	14	15	16	16	17	18	18	19	19	20	20
	V=1	germline	50	47	43	41	38	36	34	33	31	30	29	27	26	25	24	24	23	22	21	21	20
	V=4	somatic	0	9	17	24	31	36	41	46	50	54	57	60	63	66	68	71	73	75	77	78	80
	V=4	germline	50	53	57	59	62	64	66	67	69	70	71	73	74	75	76	76	77	78	79	79	80
het	V=2	somatic	0	5	9	12	15	18	21	23	25	27	29	30	32	33	34	35	36	37	38	39	40
	V=2	germline	50	49	48	47	46	45	45	44	44	43	43	42	42	42	41	41	41	41	40	40	40
	V=3	somatic	0	7	13	18	23	27	31	34	38	40	43	45	47	49	51	53	55	56	57	59	60
	V=3	germline	50	51	52	53	54	55	55	56	56	57	57	58	58	58	59	59	59	59	60	60	60
