# Supplementary material for: A computational approach to distinguish somatic vs. germline origin of genomic alterations from deep sequencing of cancer specimens without a matched normal
Source: PLoS Comput Biol. 2018 Feb 7;14(2):e1005965. doi: 10.1371/journal.pcbi.1005965 (PMC5832436; doi:10.1371/journal.pcbi.1005965)
Supplement: S2 Fig — (PDF) [file pcbi.1005965.s004.pdf]

S2 Fig. All possible SGZ prediction outcomes and an example of a cancer specimen across the genome.

(A) All possible SGZ prediction outcome ($\alpha = 0.01$).

$P(y\|S;\ AF_{somatic})$	$P(y\|G;\ AF_{germline})$	**Other conditions**	**Prediction**
$> \alpha$	$< \alpha$	NA	somatic
$\leq \alpha$	$> \alpha$	NA	germline
$\leq \alpha$	$\leq \alpha$	$f < AF_{somatic}/1.5$ and $p > 20\%$	subclonal somatic
$\leq \alpha$	$\leq \alpha$	$f \geq AF_{somatic}/1.5$ or $p \leq 20\%$	ambiguous: base on the observed data, the mutation is neither germline nor somatic. Two major causes for this scenario are: 1) The observed allele frequency f is incorrectly estimated, which is common in hard to map genomic regions such as HLA; 2) The copy number model is not correctly modelled, which is common when there is high noise in the copy number model (such as due to high GC bias).
$> \alpha$	$> \alpha$	NA	ambiguous: base on the observed data, the mutation can be both germline and somatic. This happens when the $AF_{germline}$ equals to $AF_{somatic}$ or when they are very close. An example is when the tumor content is extremely high, for a heterozygous mutation at a position with no copy number change, the $AF_{germline}$ is 0.5, and the $AF_{somatic}$ should be very close to 0.5 as well. Therefore, based on the local read depth n, and mutation allele frequency f, it is impossible to determine if this mutation is germline or somatic. More examples can be found at S1. Fig.

(B) Example of a cancer specimen across the genome. The top panel shows the log ratio profile, fitted with copy number at each segment. The Y-axis denotes log-ratio measurements of coverage obtained in test samples versus an unmatched normal control, with fitted copy levels marked by dashed lines and labeled on the right. Each point denotes a genomic region measured by the assay (blue: exon, cyan: SNP), ordered by genomic position (X-axis). Red bars indicate average log-ratio in a segment, and green lines are the model prediction. The second panel shows the corresponding minor allele frequencies at >3,500 germline SNPs, a fraction of which are polymorphic and informative. Each dot represents the MAF of a SNP measured in the assay. Each red line is the average MAF level in a segment, the green line in the same segment as fitted by the model. Regions of LOH are highlighted in red. The table at the bottom shows selected segments with LOH or chromosomal arm gain events, as well as the SGZ prediction of 2 variants corresponding to each region. An example of SGZ application to derive the variant status is provided.

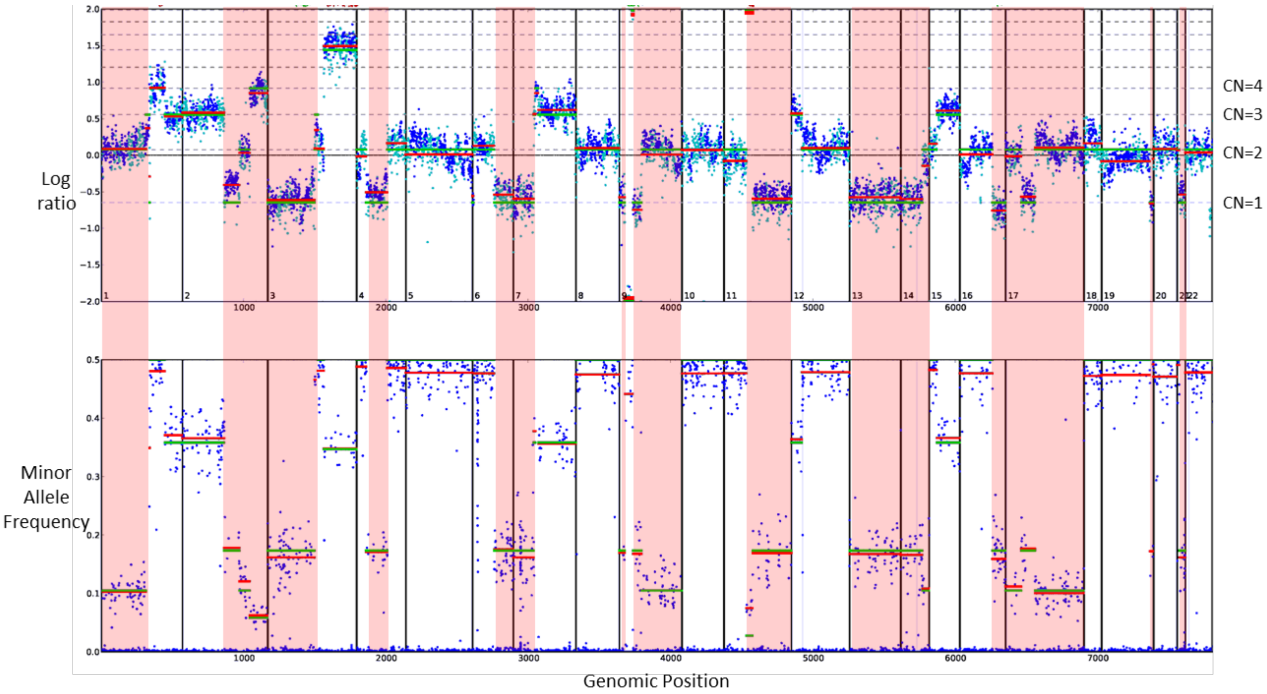

						Status of short variants					
chr	start (Mb)	end (Mb)	CN	LOH	chr arm status*	SNP	protein effect	mutation AF	depth	origin	zygosity
chr1	1	120	2	LOHx	1p_LOHx						
chr3	1	90	1	LOH1	3p_LOH1						
chr3	130	198	6	none	3q_gain						
chr13	1	115	1	LOH1	chr13_LOH1	BRCA2	D651N	67%	390	somatic	homozygous
chr17	1	8	2	LOHx	17p_LOHx	TP53	R282W	80%	686	somatic	homozygous

(Purity estimation from the copy number model fitting algorithm is 80%.)

Taking BRCA2_D651N at chr13 as an example to illustrate algorithm SGZ, i as the segment the mutation locates at, we map tumor ploidy $C_i = 1$, purity $p = 80\%$ and mutation allele frequency $f = 67\%$ to S1 Fig, predicting that the short variant is a somatic, homozygous mutation ($V_i = M_i = 1,\ V_i = C_i$ and $V_i \neq 0$).

To demonstrate the statistical prediction process, given $C_i = 1$, purity $p = 80\%$, when $V_i = C_i = 1$:

$$AF_{somatic} = \frac{pV_i}{pC_i + 2(1-p)} = \frac{0.8 * 1}{0.8 * 1 + 2 * (1 - 0.8)} = 0.67$$

$$AF_{germline} = \frac{pV_i + 1 - p}{pC_i + 2(1-p)} = \frac{0.8 * 1 + 1 - 0.8}{0.8 * 1 + 2 * (1 - 0.8)} = 0.83$$

By using coverage $n = 390$, the statistical significance of the prediction can be calculated:

$$P(y|S;\ AF_{somatic}) = bin(390 * 0.67, 390, AF_{somatic}) = 0.954 > \alpha$$

$$p(y|G;\ AF_{germline}) = bin(390 * 0.67, 390, AF_{germline}) = 2.498 \cdot 10^{-15} < \alpha$$

Where $bin(x, n, p)$ represents the binomial test. Therefore, the mutation BRCA2_D651N is predicted to be somatic, homozygous.

* A distinction between copy loss LOH (LOH1) and copy neutral LOH (LOHx) is made: LOH1 has copy number equal to one, while LOHx has copy number greater than one.
