# Supplementary material for: A computational approach to distinguish somatic vs. germline origin of genomic alterations from deep sequencing of cancer specimens without a matched normal
Source: PLoS Comput Biol. 2018 Feb 7;14(2):e1005965. doi: 10.1371/journal.pcbi.1005965 (PMC5832436; doi:10.1371/journal.pcbi.1005965)
Supplement: S3 Fig — (PDF) [file pcbi.1005965.s005.pdf]

S3 Fig. Exemplar high aneuploidy NCI-H1395 cell line with dilution with matched normal to 50% tumor purity to the advantage of SGZ using copy number model to make correct germline/somatic predictions, as compared to the basic method.

a). Copy number model plot of the exemplar sample. Taking chromosome 3 as an example, the green bars in the bottom plot is the expected allele frequency of a germline short variant within the range of chromosome 3, with expected allele frequency at 0.24, which is a significant deviation from 0.50. The complete annotation for the plot is available in S2 Fig.

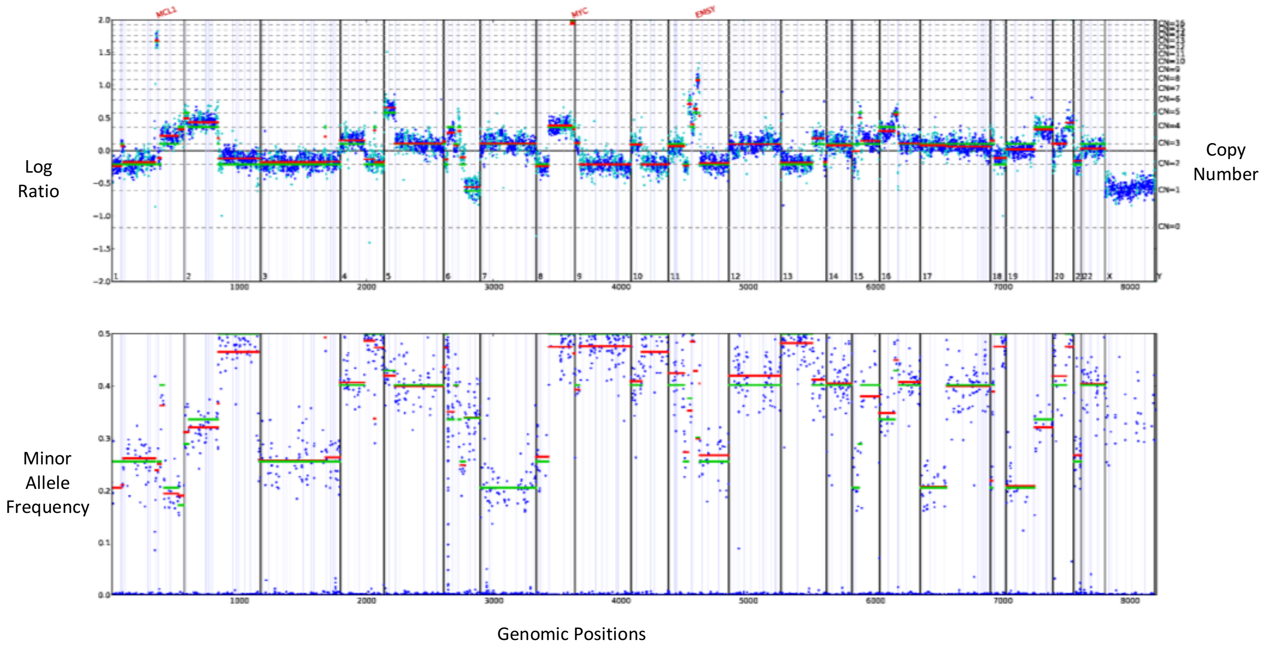

b). The complete list of short variants and corresponding germline/somatic predictions in the exemplar sample. SGZ failed to classify 1/11 mutation, namely mutation FANCA:NM_000135:c.2T>C_p.M1T, whose observed AF is within the confidence interval of both somatic mutation and germline mutation. SGZ succeeded to make correct predictions to the remaining 10/11 mutations, despite the fact that majority of the germline mutations have observed AF different from 0.50, and majority of the somatic mutations have observed AF close

to 0.50. Failing in taking copy number into consideration, Basic method only made 4/11 correct predictions.

mutation	position	AF	C	M	Gold standard	SGZ	Basic method
PBRM1:NM_018313:c.4535C>T_p.A1512V	chr3:52584478	0.24	2	0	germline	germline	somatic
FLT4:NM_002020:c.194A>T_p.Q65L	chr5:180057761	0.21	3	1	somatic	somatic	somatic
BRAF:NM_004333:c.1406G>C_p.G469A	chr7:140481402	0.52	3	0	somatic	somatic	germline
FANCG:NM_004629:c.181C>T_p.P61S	chr9:35078728	0.5	2	1	germline	germline	germline
PTCH1:NM_000264:c.3538C>G_p.P1180A	chr9:98212134	0.49	2	1	germline	germline	germline
ATM:NM_000051:c.7996A>G_p.T2666A	chr11:108204681	0.48	2	0	somatic	somatic	germline
CREBBP:NM_004380:c.5933A>G_p.N1978S	chr16:3779115	0.32	4	1	germline	germline	somatic
FANCA:NM_000135:c.2T>C_p.M1T	chr16:89883022	0.37	3	1	germline	ambiguous	somatic
DOT1L:NM_032482:c.3155G>T_p.G1052V	chr19:2222323	0.58	3	0	somatic	somatic	somatic
GNAS:NM_001077490:c.1268C>A_p.P423H	chr20:57429775	0.52	4	2	germline	germline	germline
STK11:NM_000455:c.169_169delG_p.E57fs*7	chr19:1207080	0.51	3	0	somatic	somatic	germline
