# Supplementary material for: A computational approach to distinguish somatic vs. germline origin of genomic alterations from deep sequencing of cancer specimens without a matched normal
Source: PLoS Comput Biol. 2018 Feb 7;14(2):e1005965. doi: 10.1371/journal.pcbi.1005965 (PMC5832436; doi:10.1371/journal.pcbi.1005965)
Supplement: S1 Table — (PDF) [file pcbi.1005965.s006.pdf]

**S1 Table. Somatic hotspot mutations and germline polymorphisms used for SGZ validation**

A. Somatic hotspot mutations in Fig 3A

| Gene | Mutation | Germline calls | Somatic calls |
|---|---|---|---|
| *AKT1* | E17K | 8 | 106 |
| *BRAF* | V600† | 12 | 398 |
| *BRAF* | D594† | 0 | 56 |
| *EGFR* | S768I | 0 | 16 |
| *EGFR* | L858R | 5 | 143 |
| *ERBB2* | S310F | 3 | 66 |
| *IDH1* | R132H | 13 | 144 |
| *KRAS* | G13† | 12 | 281 |
| *KRAS* | G12† | 82 | 2196 |
| *KRAS* | Q61† | 6 | 218 |
| *MAP2K1* | K57† | 0 | 22 |
| *NRAS* | G13† | 2 | 35 |
| *NRAS* | G12† | 7 | 79 |
| *NRAS* | Q61† | 20 | 279 |
| *PIK3CA* | E542† | 12 | 286 |
| *PIK3CA* | H1047† | 20 | 507 |
| *PIK3CA* | E545† | 13 | 493 |

† Includes all missense mutations of the codon

B. Germline polymorphisms in Fig 3A

| Gene | Mutation | Germline calls | Somatic calls |
|---|---|---|---|
| *GATA2* | P161A | 350 | 7 |
| *MAP3K1* | S939C | 638 | 55 |
| *NTRK1* | G18E | 365 | 3 |
| *BRCA2* | K3326* | 288 | 5 |
| *FANCF* | A186V | 275 | 2 |
| *PNRC1* | A152V | 268 | 5 |
| *GPR124* | V1146A | 244 | 10 |
| *TSC1* | K587R | 217 | 2 |
| *ZNF217* | M410V | 218 | 5 |
| *CREBBP* | N1978S | 183 | 3 |
| *BRIP1* | V193I | 179 | 2 |
| *CTCF* | P315S | 140 | 0 |
| *APC* | I1307K | 140 | 2 |
| *NOTCH2* | R91L | 141 | 6 |
| *RPTOR* | A862T | 128 | 3 |
| *ESR1* | H6Y | 125 | 3 |

| | | | |
|---|---|---|---|
| *TSHR* | V721F | 92 | 1 |
| *FAT3* | A3418S | 77 | 1 |
| *RAD52* | S346* | 59 | 1 |
| *PIK3C2G* | *1446S | 45 | 1 |
