# Supplementary material for: A computational approach to distinguish somatic vs. germline origin of genomic alterations from deep sequencing of cancer specimens without a matched normal
Source: PLoS Comput Biol. 2018 Feb 7;14(2):e1005965. doi: 10.1371/journal.pcbi.1005965 (PMC5832436; doi:10.1371/journal.pcbi.1005965)
Supplement: S2 Table — (PDF) [file pcbi.1005965.s007.pdf]

**S2 Table. List of variants in COSMIC predicted to be germline.**

| Gene | Protein change | CDS change | hg19 coordinate | dbSNP ID | Common SNP in 1000 Genomes | P-value |
|---|---|---|---|---|---|---|
| *EP300* | P925T | 2773C>A | chr22:41546158 | rs148884710 | no | 7.97E-235 |
| *VHL* | P25L | 74C>T | chr3:10183605 | rs35460768 | no | 2.99E-191 |
| *CSF1R* | V32G | 95T>G | chr5:149460542 | rs56048668 | no | 3.40E-181 |
| *APC* | I1307K | 3920T>A | chr5:112175211 | rs1801155 | no | 1.54E-159 |
| *RET* | Y791F | 2372A>T | chr10:43613908 | rs77724903 | no | 6.41E-124 |
| *MSH6* | V509A | 1526T>C | chr2:48026648 | rs63751005 | no | 2.96E-84 |
| *MLL* | L3614P | 10841T>C | chr11:118379865 | rs146191865 | yes | 7.61E-71 |
| *IL7R* | T244I | 731C>T | chr5:35874575 | rs6897932 | yes | 2.28E-60 |
| *CREBBP* | S893L | 2678C>T | chr16:3820773 | rs142047649 | no | 5.71E-47 |
| *ATM* | S978P | 2932T>C | chr11:108141988 | rs139552233 | no | 2.01E-45 |
| *CREBBP* | P858S | 2572C>T | chr16:3820879 | rs145733598 | yes | 7.78E-40 |
| *IGF1R* | R595H | 1784G>A | chr15:99456467 | rs45483397; rs56248469 | no | 1.26E-36 |
| *PAX5* | S213L | 638C>T | chr9:36966688 | rs137870876 | no | 2.40E-36 |
| *MSH2* | G322D | 965G>A | chr2:47643457 | rs4987188 | yes | 1.20E-35 |
| *PARP3* | S91N | 272G>A | chr3:51978193 | rs34224216 | yes | 1.14E-34 |
| *TBX3* | S615A | 1843T>G | chr12:115110035 | . | no | 4.10E-34 |
| *GRIN2A* | A1276G | 3827C>G | chr16:9857574 | rs145063086 | no | 5.72E-34 |
| *RET* | R114H | 341G>A | chr10:43597793 | rs76397662 | yes | 5.16E-32 |
| *CARD11* | N191S | 572A>G | chr7:2983958 | rs147264763 | no | 9.78E-32 |
| *MLL2* | P1131L | 3392C>T | chr12:49443979 | rs201623566 | no | 1.85E-31 |
| *BRCA2* | S1982fs*22 | 5946_5946delT | chr13:32914437 | rs80359550 | no | 4.90E-31 |
| *APC* | R1171C | 3511C>T | chr5:112174802 | rs201830995 | no | 3.79E-26 |
| *FLT4* | S637R | 1911C>G | chr5:180048651 | rs148898412 | no | 3.61E-25 |
| *EGFR* | P848L | 2543C>T | chr7:55259485 | rs148934350 | no | 3.42E-24 |
| *MLL2* | R1388L | 4163G>T | chr12:49441821 | rs202217665 | no | 3.25E-23 |
| *RB1* | G310E | 929G>A | chr13:48939097 | rs200844292 | no | 2.94E-21 |
| *ALK* | E1419K | 4255G>A | chr2:29416698 | rs56181542 | no | 5.56E-21 |
| *EGFR* | E282K | 844G>A | chr7:55221800 | rs199796955 | no | 2.00E-20 |
| *VHL* | P81S | 241C>T | chr3:10183772 | rs104893829 | no | 2.52E-18 |
| *CDKN2A* | I49T | 146T>C | chr9:21974681 | rs199907548 | no | 2.39E-17 |
| *FBXW7* | E192A | 575A>C | chr4:153271203 | rs201015633 | no | 4.53E-17 |
| *KDR* | N793S | 2378A>G | chr4:55964435 | rs200147160 | yes | 2.16E-15 |
| *STK11* | F298L | 894C>A | chr19:1221979 | rs199681533 | no | 2.16E-15 |
| *LRP1B* | R1072H | 3215G>A | chr2:141680638 | rs370586151 | no | 1.47E-14 |
| *AURKB* | R147W | 439C>T | chr17:8110166 | rs148133660 | no | 2.05E-14 |
| *MLH1* | R217C | 649C>T | chr3:37053562 | rs4986984 | no | 3.69E-13 |
| *PIK3C2G* | I294T | 881T>C | chr12:18443908 | . | no | 6.99E-13 |
| *BRCA2* | T1354M | 4061C>T | chr13:32912553 | rs80358656 | no | 1.85E-12 |
| *FANCA* | M160I | 480G>A | chr16:89877157 | rs200603300 | no | 1.85E-12 |
| *SETD2* | I2295M | 6885A>G | chr3:47098389 | rs150476239 | no | 1.85E-12 |
| *TP53* | R290H | 869G>A | chr17:7577069 | rs55819519 | no | 6.64E-12 |
| *MLL2* | L2610P | 7829T>C | chr12:49433724 | rs200998047 | no | 1.76E-11 |
| *VHL* | R200W | 598C>T | chr3:10191605 | rs28940298 | no | 3.33E-11 |
| *AXL* | V289M | 865G>A | chr19:41743930 | rs141302305 | no | 6.31E-11 |
| *PTEN* | A79T | 235G>A | chr10:89690828 | rs202004587 | no | 1.20E-10 |
| *BRCA1* | C61G | 181T>G | chr17:41258504 | rs28897672 | no | 1.67E-10 |
| *FANCM* | R658* | 1972C>T | chr14:45636336 | rs368728266 | no | 1.67E-10 |
| *TP53* | N235S | 704A>G | chr17:7577577 | rs144340710 | no | 3.16E-10 |
| *BRCA2* | A938fs*21 | 2808_2811delACAA | chr13:32911299 | rs80359352 | no | 3.16E-10 |
| *PARP1* | A625T | 1873G>A | chr1:226564877 | . | no | 3.16E-10 |
| *FAT3* | T3929M | 11786C>T | chr11:92600034 | . | no | 1.59E-09 |
| *AXL* | V744M | 2230G>A | chr19:41763458 | rs372169583 | no | 1.59E-09 |
| *FANCM* | S1276L | 3827C>T | chr14:45645784 | . | no | 1.51E-08 |

| | | | | | | |
|---|---|---|---|---|---|---|
| *CREBBP* | A467T | 1399G>A | chr16:3832859 | rs202225861 | no | 1.51E-08 |
| *MLH1* | R325Q | 974G>A | chr3:37061890 | rs63750268 | no | 1.51E-08 |
| *CDKN2A* | A121T | 361G>A | chr9:21971040 | rs199888003 | no | 1.51E-08 |
| *PTCH1* | T416S | 1247C>G | chr9:98240437 | rs201174718 | no | 2.86E-08 |
| *EPHB1* | V322I | 964G>A | chr3:134851558 | . | no | 3.87E-08 |
| *NRAS* | P185S | 553C>T | chr1:115251173 | rs374061873 | no | 1.43E-07 |
| *ASXL1* | V1367I | 4099G>A | chr20:31024614 | rs147456014 | no | 1.43E-07 |
| *RET* | V804M | 2410G>A | chr10:43614996 | rs79658334 | no | 1.43E-07 |
| *FGFR1* | T141R | 422C>G | chr8:38285890 | rs200482627 | no | 1.43E-07 |
| *PMS2* | V415M | 1243G>A | chr7:6027153 | rs138387687 | no | 2.71E-07 |
| *ATR* | R1082H | 3245G>A | chr3:142266679 | rs146504354 | no | 2.71E-07 |
| *KDM2B* | T28fs*8 | 82_83delAC | chr12:122018733 | . | no | 9.74E-07 |
| *MLL* | K2658N | 7974G>C | chr11:118374590 | rs142807735 | no | 1.36E-06 |
| *RET* | E511K | 1531G>A | chr10:43607555 | rs201553718 | yes | 2.58E-06 |
| *CDKN2A* | A57V | 170C>T | chr9:21971188 | rs372266620 | no | 2.58E-06 |
| *CDH1* | S838G | 2512A>G | chr16:68867265 | rs121964872 | no | 2.58E-06 |
