# Supplementary material for: A computational approach to distinguish somatic vs. germline origin of genomic alterations from deep sequencing of cancer specimens without a matched normal
Source: PLoS Comput Biol. 2018 Feb 7;14(2):e1005965. doi: 10.1371/journal.pcbi.1005965 (PMC5832436; doi:10.1371/journal.pcbi.1005965)
Supplement: S3 Table — (PDF) [file pcbi.1005965.s008.pdf]

S3 Table. Summary of 84 samples from 30 non-small cell lung & colon cancer patients.

Patient ID	site of primary tumor	Sample type	gender	median coverage	computational purity assessment (%)
1	Lung	normal	M	830	NA
1	Lung	primary	M	909	53
1	Lung	metastatic	M	891	50
2	Lung	normal	F	943	NA
2	Lung	primary	F	852	30
2	Lung	metastatic	F	832	20
3	Lung	normal	M	832	NA
3	Lung	primary	M	845	32
3	Lung	metastatic	M	729	47
4	Lung	normal	M	827	NA
4	Lung	primary	M	583	40
4	Lung	metastatic	M	782	24
5	Lung	normal	M	913	NA
5	Lung	primary	M	893	30
5	Lung	metastatic	M	932	30
6	Lung	normal	M	845	NA
6	Lung	primary	M	668	40
6	Lung	metastatic	M	819	50
7	Lung	normal	M	811	NA
7	Lung	primary	M	798	50
7	Lung	metastatic	M	851	44
8	Lung	normal	M	555	NA
8	Lung	primary	M	731	65
8	Lung	metastatic	M	814	20
9	Lung	normal	M	875	NA
9	Lung	primary	M	758	42
9	Lung	metastatic	M	643	45
10	Lung	normal	M	723	NA
10	Lung	primary	M	690	66
10	Lung	metastatic	M	390	70
11	Lung	normal	M	774	NA
11	Lung	primary	M	584	30
11	Lung	metastatic	M	299	40
12	Lung	normal	M	756	NA
12	Lung	primary	M	795	40
12	Lung	metastatic	M	926	60
13	Colon	normal	F	1001	NA
13	Colon	primary	F	1097	30
13	Colon	metastatic	F	1095	50
14	Colon	normal	M	980	NA
14	Colon	metastatic	M	998	67
15	Colon	normal	M	595	NA
15	Colon	metastatic	M	890	20
16	Colon	primary	F	587	20
16	Colon	normal	F	991	NA
16	Colon	metastatic	F	1025	49
17	Colon	primary	M	1017	80
17	Colon	normal	M	797	NA
17	Colon	metastatic	M	803	68
18	Colon	normal	M	786	NA
18	Colon	primary	M	719	40
18	Colon	metastatic	M	913	69

19	Colon	normal	M	737	NA
19	Colon	primary	M	992	20
19	Colon	metastatic	M	864	30
20	Colon	normal	M	984	NA
21	Colon	normal	F	742	NA
21	Colon	primary	F	878	50
22	Colon	normal	M	1107	NA
22	Colon	primary	M	963	34
22	Colon	metastatic	M	882	40
23	Colon	normal	M	812	NA
23	Colon	primary	M	1087	70
23	Colon	metastatic	M	802	90
24	Colon	normal	M	795	NA
24	Colon	primary	M	601	22
24	Colon	metastatic	M	702	58
25	Colon	normal	M	837	NA
25	Colon	primary	M	855	70
25	Colon	metastatic	M	1112	56
26	Colon	normal	M	891	NA
26	Colon	primary	M	980	56
26	Colon	metastatic	M	764	20
27	Rectum	normal	M	917	NA
27	Rectum	primary	M	844	50
27	Rectum	metastatic	M	812	30
28	Gastric	normal	M	786	NA
28	Gastric	primary	M	793	50
28	Gastric	metastatic	M	661	70
29	Gastric	normal	M	802	NA
29	Gastric	primary	M	963	50
30	Gastric	normal	M	920	NA
30	Gastric	primary	M	838	40
30	Gastric	metastatic	M	777	40
