# Supplementary material for: A computational approach to distinguish somatic vs. germline origin of genomic alterations from deep sequencing of cancer specimens without a matched normal
Source: PLoS Comput Biol. 2018 Feb 7;14(2):e1005965. doi: 10.1371/journal.pcbi.1005965 (PMC5832436; doi:10.1371/journal.pcbi.1005965)
Supplement: S4 Table — (PDF) [file pcbi.1005965.s009.pdf]

S4 Table. Mutations from cell line dataset that were detected by our pipeline and used for SGZ validation.

Cell line ID	Mutation	Genomic Position	Reference mutation status[†††]
HCC-1937	RUNX1:NM_001754:c.1106C>T_p.S369L	chr21:36164769	Germline
HCC-1937	DIS3:NM_001128226:c.80A>G_p.D27G	chr13:73355891	Germline
HCC-1937	BARD1:NM_000465:c.1071_1091delACCATTGCCTGAATGTTCTTC_p.P358_S364del[††]	chr2:215645506	Germline
HCC-1937	MLL2:NM_003482:c.1952C>T_p.S651L	chr12:49445514	Germline
HCC-1937	ARID2:NM_152641:c.3217C>T_p.P1073S	chr12:46245123	Germline
HCC-1937	GSK3B:NM_002093:c.930T>A_p.H310Q	chr3:119585455	Germline
HCC-1937	BRCA1:NM_007294:c.5263_5264insC_p.Q1756fs*74	chr17:41209082	Germline
HCC-1937	RB1:NM_000321:c.2222_2325del178_p.R741fs*19[†]	chr13:49039143	Somatic
HCC-1937	ARFRP1:NM_003224:c.322C>G_p.L108V	chr20:62333512	Somatic
HCC-1937	NTRK2:NM_006180:c.1591C>G_p.Q531E	chr9:87482304	Somatic
HCC-1937	TP53:NM_000546:c.916C>T_p.R306*	chr17:7577022	Somatic
HCC-1937	LRP1B:NM_018557:c.1790-1G>C	chr2:141777672	Somatic
HCC-1937	LRP1B:NM_018557:c.8593T>A_p.C2865S	chr2:141260601	Somatic
HCC-1937	NF1:NM_001042492:c.2099C>G_p.T700S	chr17:29553550	Somatic
HCC-1954	TET2:NM_001127208:c.1381C>T_p.Q461*	chr4:106156480	Germline
HCC-1954	FANCA:NM_000135:c.4036G>A_p.A1346T	chr16:89805672	Germline
HCC-1954	TSC2:NM_000548:c.2348C>G_p.T783S	chr16:2122977	Germline
HCC-1954	BARD1:NM_000465:c.1071_1091delACCATTGCCTGAATGTTCTTC_p.P358_S364del[††]	chr2:215645506	Germline
HCC-1954	EPHA3:NM_005233:c.872C>A_p.P291Q	chr3:89390123	Germline
HCC-1954	NBN:NM_002485:c.758C>T_p.T253I	chr8:90982730	Germline
HCC-1954	MLL2:NM_003482:c.11083C>T_p.P3695S	chr12:49427405	Germline
HCC-1954	EPHB1:NM_004441:c.2942C>T_p.T981M	chr3:134977949	Germline
HCC-1954	DIS3:NM_001128226:c.1457G>T_p.G486V	chr13:73345991	Germline
HCC-1954	SETD2:NM_014159:c.6686T>G_p.V2229G	chr3:47098588	Germline
HCC-1954	NOTCH1:NM_017617:c.3644G>A_p.G1215D	chr9:139401425	Somatic
HCC-1954	PIK3CA:NM_006218:c.3140A>G_p.H1047R	chr3:178952085	Somatic
HCC-1954	TP53:NM_000546:c.488A>G_p.Y163C	chr17:7578442	Somatic
HCC-1954	PARP4:NM_006437:c.3509C>T_p.T1170I	chr13:25016762	Somatic
HCC-1954	BRCA1:NM_007294:c.5425_5426delGT_p.V1809fs*20	chr17:41199700	Somatic
HCC-1954	APC:NM_000038:c.6998G>A_p.R2333K	chr5:112178289	Somatic
NCI-H1395	PBRM1:NM_018313:c.4535C>T_p.A1512V	chr3:52584478	Germline
NCI-H1395	GNAS:NM_001077490:c.1268C>A_p.P423H	chr20:57429775	Germline
NCI-H1395	FANCA:NM_000135:c.2T>C_p.M1T	chr16:89883022	Germline
NCI-H1395	PTCH1:NM_000264:c.3538C>G_p.P1180A	chr9:98212134	Germline
NCI-H1395	FANCG:NM_004629:c.181C>T_p.P61S	chr9:35078728	Germline
NCI-H1395	CREBBP:NM_004380:c.5933A>G_p.N1978S	chr16:3779115	Germline
NCI-H1395	STK11:NM_000455:c.169_169delG_p.E57fs*7	chr19:1207080	Somatic
NCI-H1395	ATM:NM_000051:c.7996A>G_p.T2666A	chr11:108204681	Somatic

NCI-H1395	FLT4:NM_002020:c.194A>T_p.Q65L	chr5:180057761	Somatic
NCI-H1395	DOT1L:NM_032482:c.3155G>T_p.G1052V	chr19:2222323	Somatic
NCI-H1395	BRAF:NM_004333:c.1406G>C_p.G469A	chr7:140481402	Somatic
NCI-H1395	DNMT3A:NM_022552:c.1226G>A_p.W409*	chr2:25469542	Somatic
NCI-H1395	CDKN2A:NM_000077:c.1_8delGGCGGCGGGGAGCAGCATGGAGCC_p.M1fs*11	chr9:21974818	Somatic

†Full annotation to short variant RB1:NM_000321:c.2222_2325del178_p.R741fs*19:frameshift: RB1:NM_000321:c.2222_2325delGTGTTTTGATCAAAGAAGAGGAGTATGATTCTATTAT AGTATTCTATAACTCGGTCTTCATGCAGAGACTGAAAACAAATATTTTGCAGTATGC TTCCACCAGGGTAGGTCAAAAGTATCCTTTGATTGGAAAAATCTAATGTAATGGGTC CACCAAAACATTAAATAAATAATCTAC_p.R741fs*19:frameshift.

†† Mutation BARD1:NM_000465:c.1071_1091delACCATTGCCTGAATGTTCTTC_p.P358_S364del:nonframeshift in cell line HCC-1937 and HCC-1954 is in a highly repetitive region and allele frequency estimation of this short variant is always poor in our pipeline. In this dataset with relatively small number of short variants, we excluded this short variant in analysis to avoid severe degradation in performance evaluation.

††† Reference mutation status was obtained by sequencing and comparing the 100% tumor content cell line with its matched normal.
