# Supplementary material for: A computational approach to distinguish somatic vs. germline origin of genomic alterations from deep sequencing of cancer specimens without a matched normal
Source: PLoS Comput Biol. 2018 Feb 7;14(2):e1005965. doi: 10.1371/journal.pcbi.1005965 (PMC5832436; doi:10.1371/journal.pcbi.1005965)
Supplement: S5 Table — (PDF) [file pcbi.1005965.s010.pdf]

S5 Table. Mutations incorrectly classified by the basic method and correctly classified by SGZ in regions of copy number change in the cell line dataset. As shown in the table, out of a total number of 184 mutations that are correctly classified by SGZ in the dataset, 63 of them are incorrectly classified by the basic method due to local copy number deviation from 2 and/or zygosity deviation from balanced heterozygosity (defined as M divided by C equal to 0.5).

Sample ID/ Tumor purity	Cell line ID	Mutation	AF	depth	C	M	gold standard	SGZ prediction	Basic method prediction
1/10%	HCC-1937	RUNX1:NM_001754:c.1106C>T_p.S369L	0.56	854	2	0	germline	germline	somatic
1/10%	HCC-1937	DIS3:NM_001128226:c.80A>G_p.D27G	0.61	468	2	0	germline	germline	somatic
1/10%	HCC-1937	MLL2:NM_003482:c.1952C>T_p.S651L	0.59	1049	1	0	germline	germline	somatic
1/10%	HCC-1937	ARID2:NM_152641:c.3217C>T_p.P1073S	0.54	1269	1	0	germline	germline	somatic
1/10%	HCC-1937	GSK3B:NM_002093:c.930T>A_p.H310Q	0.58	856	2	0	germline	germline	somatic
1/10%	HCC-1937	BRCA1:NM_007294:c.5263_5264insC_p.Q1756fs*74	0.57	824	2	0	germline	germline	somatic
2/10%	NCI-H1395	PBRM1:NM_018313:c.4535C>T_p.A1512V	0.39	542	3	1	germline	germline	somatic
2/10%	NCI-H1395	FANCA:NM_000135:c.2T>C_p.M1T	0.43	228	3	1	germline	germline	somatic
2/10%	NCI-H1395	CREBBP:NM_004380:c.5933A>G_p.N1978S	0.44	526	3	1	germline	germline	somatic
3/20%	HCC-1937	RUNX1:NM_001754:c.1106C>T_p.S369L	0.62	525	3	0	germline	germline	somatic
3/20%	HCC-1937	DIS3:NM_001128226:c.80A>G_p.D27G	0.75	324	6	0	germline	germline	somatic
3/20%	HCC-1937	MLL2:NM_003482:c.1952C>T_p.S651L	0.66	749	3	0	germline	germline	somatic
3/20%	HCC-1937	ARID2:NM_152641:c.3217C>T_p.P1073S	0.61	757	4	1	germline	germline	somatic
3/20%	HCC-1937	GSK3B:NM_002093:c.930T>A_p.H310Q	0.7	540	4	0	germline	germline	somatic
4/20%	HCC-1954	TET2:NM_001127208:c.1381C>T_p.Q461*	0.43	687	5	2	germline	germline	somatic
4/20%	HCC-1954	EPHA3:NM_005233:c.872C>A_p.P291Q	0.42	551	4	2	germline	germline	somatic
4/20%	HCC-1954	SETD2:NM_014159:c.6686T>G_p.V2229G	0.46	969	4	2	germline	germline	somatic
5/20%	NCI-H1395	PBRM1:NM_018313:c.4535C>T_p.A1512V	0.34	587	2	0	germline	germline	somatic
5/20%	NCI-H1395	FANCA:NM_000135:c.2T>C_p.M1T	0.39	271	3	1	germline	germline	somatic
5/20%	NCI-H1395	CREBBP:NM_004380:c.5933A>G_p.N1978S	0.42	651	4	1	germline	germline	somatic
6/30%	HCC-1937	RUNX1:NM_001754:c.1106C>T_p.S369L	0.7	751	3	0	germline	germline	somatic
6/30%	HCC-1937	DIS3:NM_001128226:c.80A>G_p.D27G	0.8	443	7	0	germline	germline	somatic
6/30%	HCC-1937	MLL2:NM_003482:c.1952C>T_p.S651L	0.7	895	3	0	germline	germline	somatic
6/30%	HCC-1937	ARID2:NM_152641:c.3217C>T_p.P1073S	0.61	1073	4	1	germline	germline	somatic
6/30%	HCC-1937	GSK3B:NM_002093:c.930T>A_p.H310Q	0.75	752	5	0	germline	germline	somatic
6/30%	HCC-1937	BRCA1:NM_007294:c.5263_5264insC_p.Q1756fs*74	0.69	668	4	0	germline	germline	somatic
6/30%	HCC-1937	TP53:NM_000546:c.916C>T_p.R306*	0.5	916	4	0	somatic	somatic	germline
7/30%	HCC-1954	TET2:NM_001127208:c.1381C>T_p.Q461*	0.41	454	3	1	germline	germline	somatic
7/30%	HCC-1954	SETD2:NM_014159:c.6686T>G_p.V2229G	0.46	747	3	1	germline	germline	somatic
8/30%	NCI-H1395	FANCA:NM_000135:c.2T>C_p.M1T	0.39	99	3	1	germline	germline	somatic
9/40%	HCC-1937	RUNX1:NM_001754:c.1106C>T_p.S369L	0.75	728	3	0	germline	germline	somatic

9/40%	HCC-1937	DIS3:NM_001128226:c.80A>G_p.D27G	0.81	497	6	0	germline	germline	somatic
9/40%	HCC-1937	MLL2:NM_003482:c.1952C>T_p.S651L	0.75	877	3	0	germline	germline	somatic
9/40%	HCC-1937	ARID2:NM_152641:c.3217C>T_p.P1073S	0.64	1044	4	1	germline	germline	somatic
9/40%	HCC-1937	GSK3B:NM_002093:c.930T>A_p.H310Q	0.8	835	5	0	germline	germline	somatic
10/40%	HCC-1954	FANCA:NM_000135:c.4036G>A_p.A1346T	0.46	617	4	2	germline	germline	somatic
10/40%	HCC-1954	EPHA3:NM_005233:c.872C>A_p.P291Q	0.44	372	3	1	germline	germline	somatic
10/40%	HCC-1954	EPHB1:NM_004441:c.2942C>T_p.T981M	0.44	551	3	1	germline	germline	somatic
11/40%	NCI-H1395	PBRM1:NM_018313:c.4535C>T_p.A1512V	0.29	319	2	0	germline	germline	somatic
11/40%	NCI-H1395	CREBBP:NM_004380:c.5933A>G_p.N1978S	0.31	423	4	1	germline	germline	somatic
11/40%	NCI-H1395	DOT1L:NM_032482:c.3155G>T_p.G1052V	0.49	394	3	0	somatic	somatic	germline
11/40%	NCI-H1395	BRAF:NM_004333:c.1406G>C_p.G469A	0.45	327	3	0	somatic	somatic	germline
12/50%	HCC-1937	RUNX1:NM_001754:c.1106C>T_p.S369L	0.78	457	3	0	germline	germline	somatic
12/50%	HCC-1937	DIS3:NM_001128226:c.80A>G_p.D27G	0.89	311	7	0	germline	germline	somatic
12/50%	HCC-1937	MLL2:NM_003482:c.1952C>T_p.S651L	0.8	752	3	0	germline	germline	somatic
12/50%	HCC-1937	GSK3B:NM_002093:c.930T>A_p.H310Q	0.86	589	5	0	germline	germline	somatic
13/50%	HCC-1954	FANCA:NM_000135:c.4036G>A_p.A1346T	0.44	787	6	3	germline	germline	somatic
13/50%	HCC-1954	EPHB1:NM_004441:c.2942C>T_p.T981M	0.39	754	4	1	germline	germline	somatic
13/50%	HCC-1954	SETD2:NM_014159:c.6686T>G_p.V2229G	0.42	963	4	1	germline	germline	somatic
14/50%	NCI-H1395	PBRM1:NM_018313:c.4535C>T_p.A1512V	0.24	551	2	0	germline	germline	somatic
14/50%	NCI-H1395	CREBBP:NM_004380:c.5933A>G_p.N1978S	0.32	798	4	1	germline	germline	somatic
14/50%	NCI-H1395	STK11:NM_000455:c.169_169delG_p.E57fs*7	0.51	941	3	0	somatic	somatic	germline
14/50%	NCI-H1395	ATM:NM_000051:c.7996A>G_p.T2666A	0.48	399	2	0	somatic	somatic	germline
14/50%	NCI-H1395	BRAF:NM_004333:c.1406G>C_p.G469A	0.52	484	3	0	somatic	somatic	germline
15/75%	HCC-1937	RUNX1:NM_001754:c.1106C>T_p.S369L	0.89	523	3	0	germline	germline	somatic
15/75%	HCC-1937	MLL2:NM_003482:c.1952C>T_p.S651L	0.9	837	3	0	germline	germline	somatic
15/75%	HCC-1937	GSK3B:NM_002093:c.930T>A_p.H310Q	0.95	661	5	0	germline	germline	somatic
16/75%	HCC-1954	EPHB1:NM_004441:c.2942C>T_p.T981M	0.38	503	4	1	germline	germline	somatic
16/75%	HCC-1954	SETD2:NM_014159:c.6686T>G_p.V2229G	0.35	676	4	1	germline	germline	somatic
17/75%	NCI-H1395	PBRM1:NM_018313:c.4535C>T_p.A1512V	0.15	496	2	0	germline	germline	somatic
17/75%	NCI-H1395	FANCA:NM_000135:c.2T>C_p.M1T	0.37	222	3	1	germline	germline	somatic
17/75%	NCI-H1395	CREBBP:NM_004380:c.5933A>G_p.N1978S	0.27	700	4	1	germline	germline	somatic

AF: observed allele frequency

depth: local median depth

C: local copy number

M: local copy number of the minor allele
