# Supplementary material for: A computational approach to distinguish somatic vs. germline origin of genomic alterations from deep sequencing of cancer specimens without a matched normal
Source: PLoS Comput Biol. 2018 Feb 7;14(2):e1005965. doi: 10.1371/journal.pcbi.1005965 (PMC5832436; doi:10.1371/journal.pcbi.1005965)
Supplement: S1 Note — (PDF) [file pcbi.1005965.s011.pdf]

S1 Note. Equivalence of a subset of SGZ solutions to copy number model fitting.

Given a copy number model with tumor ploidy Ψ, purity p, copy number C_i, minor allele count M_i and mutational allele count V_i (M_i or $C_i - M_i$), the expected log-ratio level lr_i and allele frequency level f_i at genomic segment i are

$$lr_i = \log_2 \frac{pC_i + 2(1-p)}{p\Psi + 2(1-p)}$$

$$f_{i\,germline} = \frac{pV_i + (1-p)}{pC_i + 2(1-p)}$$

$$f_{i\,somatic} = \frac{pV_i}{pC_i + 2(1-p)}$$

There exists a family of models that share the same log-ratio level and minor allele frequency level, thus considered as equivalent models of the base model. Any model in a family with ploidy consistent with known cancer biology could potentially be reported as the optimal model by our pipeline.

> **Lemma.** Assume we have a copy number model with tumor ploidy Ψ, purity p, with log-ratios and germline and somatic allele frequencies as defined above. Then a model with tumor ploidy $2^k\Psi$, purity $p/(2^k(1-p)+p)$, copy number levels 2^kC_i, minor allele counts 2^kM_i and mutational allele frequency 2^kV_i will yield the same expected log-ratios and germline and somatic allele frequencies.

Proof:

$$lr_i = \log_2 \frac{\frac{p}{2^k(1-p)+p} 2^k C_i + 2\left(1 - \frac{p}{2^k(1-p)+p}\right)}{\frac{p}{2^k(1-p)+p} 2^k \Psi + 2\left(1 - \frac{p}{2^k(1-p)+p}\right)}$$
$$= \log_2 \frac{p * 2^k C_i + 2(2^k(1-p)+p-p)}{p * 2^k \Psi + 2(2^k(1-p)+p-p)} = \log_2 \frac{pC_i + 2(1-p)}{p\Psi + 2(1-p)}$$

$$f_{i\,germline} = \frac{\frac{p}{2^k(1-p)+p} 2^k V_i + \left(1 - \frac{p}{2^k(1-p)+p}\right)}{\frac{p}{2^k(1-p)+p} 2^k C_i + 2\left(1 - \frac{p}{2^k(1-p)+p}\right)}$$
$$= \frac{p * 2^k V_i + 2^k(1-p)+p-p}{p * 2^k C_i + 2(2^k(1-p)+p-p)} = \frac{pV_i + (1-p)}{pC_i + 2(1-p)}$$

$$f_{i\,somatic} = \frac{\frac{p}{2^k(1-p)+p} 2^k V_i}{\frac{p}{2^k(1-p)+p} 2^k C_i + 2\left(1 - \frac{p}{2^k(1-p)+p}\right)} = \frac{p * 2^k V_i}{p * 2^k C_i + 2(2^k(1-p)+p-p)}$$
$$= \frac{pV_i}{pC_i + 2(1-p)}$$

Hence, all the expected lr_i, $f_{i\,germline}$ and $f_{i\,somatic}$ remain unchanged from the base model. Therefore, models from this family are equivalent with respect to SGZ prediction of germline/somatic and homozygous/not in tumor status (although more levels of heterozygosity are possible in higher ploidy models), showing robustness of SGZ algorithm to different models within this family.

Example. Below is an example of 2 distinct models from the same family.

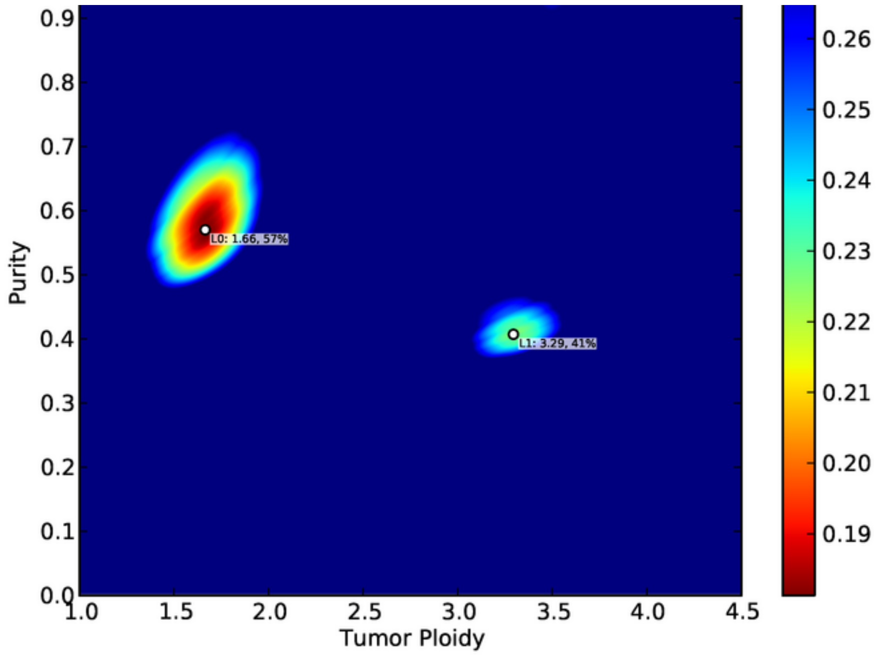

S1 Note Fig 1. Heatmap of the mean-squared error between the measured and expected copy numbers over a grid of different tumor purity and ploidy for the example sample. The model estimation L0 and L1 (double in ploidy) belong to an equivalence family. The corresponding CN profiles for model L0 and L1 is shown in S1 Note Fig 2.

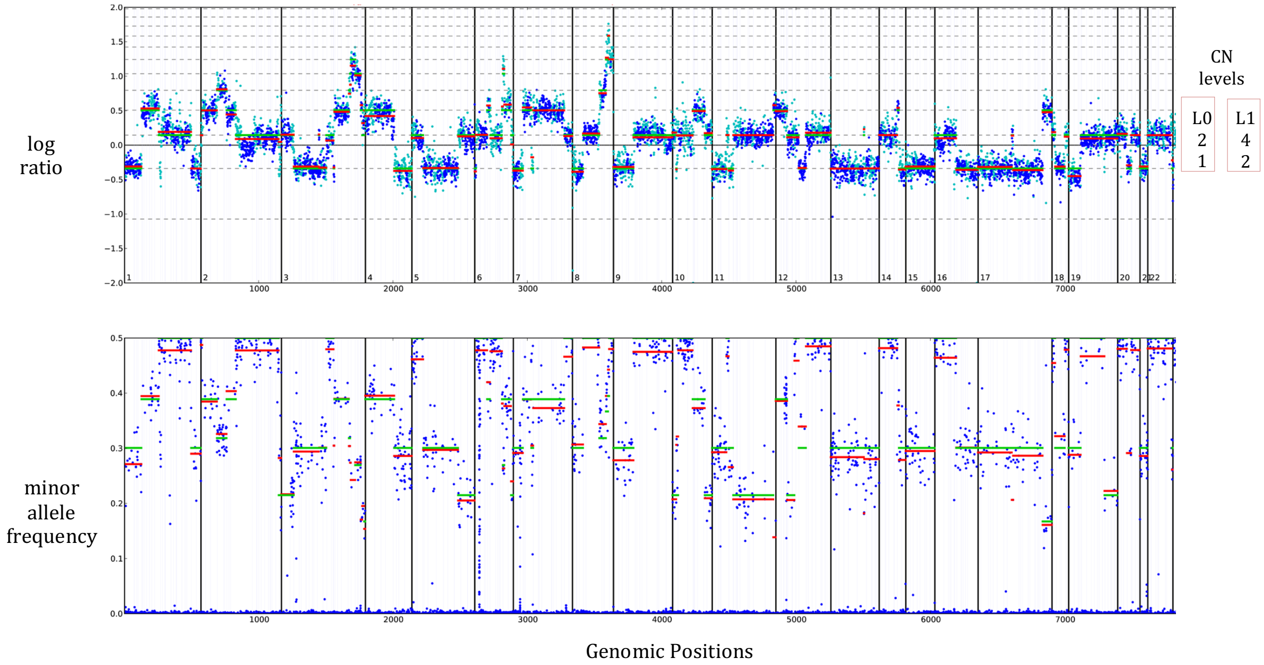

S1 Note 1 Fig 2. Copy number profile for the exemplar sample. L0 is a diploid model and L1 is the tetraploid model within the same family. The only difference is the assignment in copy level, as indicated in the top plot.
